# Adipocytes Are the Control Tower That Manages Adipose Tissue Immunity by Regulating Lipid Metabolism

**DOI:** 10.3389/fimmu.2020.598566

**Published:** 2021-01-28

**Authors:** Jeu Park, Jee Hyung Sohn, Sang Mun Han, Yoon Jeong Park, Jin Young Huh, Sung Sik Choe, Jae Bum Kim

**Affiliations:** ^1^ National Creative Research Initiatives Center for Adipocyte Structure and Function, Seoul National University, Seoul, South Korea; ^2^ Institute of Molecular Biology and Genetics, Seoul National University, Seoul, South Korea; ^3^ School of Biological Sciences, Seoul National University, Seoul, South Korea

**Keywords:** adipocytes, lipid metabolite, invariant natural killer cell, adipose tissue remodeling, adipose tissue inflammation

## Abstract

Accumulating evidence reveals that adipose tissue is an immunologically active organ that exerts multiple impacts on the regulation of systemic energy metabolism. Adipose tissue immunity is modulated by the interactions between adipocytes and various immune cells. Nevertheless, the underlying mechanisms that control inter-cellular interactions between adipocytes and immune cells in adipose tissue have not been thoroughly elucidated. Recently, it has been demonstrated that adipocytes utilize lipid metabolites as a key mediator to initiate and mediate diverse adipose tissue immune responses. Adipocytes present lipid antigens and secrete lipid metabolites to determine adipose immune tones. In addition, the interactions between adipocytes and adipose immune cells are engaged in the control of adipocyte fate and functions upon metabolic stimuli. In this review, we discuss an integrated view of how adipocytes communicate with adipose immune cells using lipid metabolites. Also, we briefly discuss the newly discovered roles of adipose stem cells in the regulation of adipose tissue immunity.

## Introduction

Adipose tissue is a specific type of loose connective tissues present in various anatomical locations. For energy homeostasis and survival, adipose tissue contributes to numerous physiological roles: it provides structural support and protective padding for major organs, it serves as an insulating layer that prevents cutaneous heat loss, it stores extra energy source for longer periods of fasting, and it is a dynamic endocrine system crucial in the regulation of energy homeostasis (1). Among the various cell types residing in adipose tissue, adipocytes are the major cell type that is specialized to synthesize and store large globules of fat (2). When energy level is low, adipocytes break down stored lipid metabolites into fatty acids and glycerol and release them into circulation, which are used for fuels in most organs. This function of adipocytes enables adipose tissue to function as the major energy reservoir. Moreover, adipocytes act as a key component of endocrine activity through secreting a variety of signaling molecules such as adipokines, lipokines, and exosomes (3). These adipocyte-derived factors are involved in the maintenance of systemic energy homeostasis through crosstalk with other tissues such as muscle, liver, and brain (2).

Adipose tissue harbors diverse innate and adaptive immune cells. Dynamic interactions between these innate and adaptive immune cells are closely associated with alterations of adipose tissue function and integrity upon metabolic changes (4–6). For example, adipose tissue immunity shifts toward pro-inflammatory state in response to chronic energy surplus such as obesity, leading to dysregulation of adipose tissue homeostasis (7–10). Among various adipose immune cells, adipose tissue macrophages (ATMs) occupy about 50% and are largely classified into pro-inflammatory M1-type and anti-inflammatory M2-macrophages (11, 12). In obesity, M1-type macrophages are abundantly accumulated and secrete pro-inflammatory molecules such as tumor necrosis factor (TNF)-α, nitric oxide (NO), and interleukin (IL)-6 (13–15). In addition, neutrophil, Th1, Th17, CD8 T cells, and group 1 innate lymphoid cell (ILC1) secrete pro-inflammatory cytokines including interferon (IFN)-*γ*, IL-6, and IL-17 (16, 17). These pro-inflammatory molecules suppress insulin action in adipocytes by inhibiting phosphorylation of insulin receptor and insulin receptor substrate 1, which provokes insulin resistance. On the other hand, there are numerous anti-inflammatory immune cells that downregulate pro-inflammatory responses, improving insulin sensitivity in adipose tissue. Eosinophil, regulatory T cell (Treg), invariant natural killer T (iNKT), and group 2 innate lymphoid cell (ILC2) stimulate to polarize macrophages towards anti-inflammatory M2-type macrophages through secretion of Th2 type cytokines, including IL-4, IL-5, IL-10, and IL-13, attenuating adipose inflammatory responses and improving insulin sensitivity (11).

Recently, emerging evidence indicates that adipocyte-derived lipid metabolites would function as a crucial regulator of adipose tissue immunity (18–21). In obese adipocytes, aberrant lipid metabolism promotes lipid spillover, which activates NF-κB pathways in ATMs and consequently induces TNF-α secretion (22). Also, dysregulation of lipokines and lipid antigens is manifested in dysfunctional adipocytes, which has been linked to changes in characteristics of adaptive immune cells in adipose tissue. It has been recently shown that adipocyte-derived lipid antigens could alter inter-cellular interactions between innate and adaptive immune cells, followed by alterations of function and fate of adipocytes (23). Despite the close association of lipid metabolism in adipocytes with adipose tissue immunity has been reported for over a decade, the molecular mediators and mechanisms linking adipocyte-derived lipid metabolites to adipose tissue immunity remain poorly understood. In previous reviews, the importance of the crosstalk between innate and adaptive immune cells in adipose tissue on energy metabolism has been well addressed (1, 11, 12). Thus, in this review, we cover the processes by which adipocytes communicate with adipose immune cells using lipid metabolites. Furthermore, we discuss the new concept that adipocytes cooperate with adipose immune cells to protect adipose tissue integrity from metabolic stresses. In addition, we briefly propose the novel roles of adipocyte stem cells in the regulation of adipose tissue immunity.

## Immunomodulatory Roles of Adipocytes Using Lipid Antigens

There are distinct types of immune cells that recognize lipid antigens. These immune cells, such as iNKT cells and *γδ* T cells, rapidly respond to changes of lipid metabolism through sensing lipid antigens loaded on antigen presenting cells (APCs). It has been reported that iNKT cells and *γδ* T cells are abundantly present in adipose tissue and actively interact with adipocytes, contributing to the regulation of systemic energy metabolism (24–27). For example, in obesity, adipose iNKT cells are activated by adipocyte-derived lipid antigens and modulate the interaction between innate and adaptive immune cells (24, 28, 29). Moreover, activation of iNKT cells by hypertrophic adipocyte-derived lipid antigens stimulates adipocyte turnover in obesity, contributing to adipose tissue remodeling (23). Similarly, *γδ* T cells regulate adipose tissue immune responses and adipocyte functions (26, 27, 30). Given that *γδ* T cells recognize CD1-loaded lipid antigens, it has been suggested that adipocytes would control *γδ* T cell activity (31, 32). In this section, we discuss detailed mechanisms by which adipocytes regulate adipose tissue immune cells *via* lipid antigen presentation.

### Lipid Antigen Presentation

In adipose tissue, there are several APCs such as dendritic cells, macrophages, B cells, and adipocytes (24, 25, 33). It has been demonstrated that adipocytes highly express MHC-I like protein, CD1d, and present lipid antigens (24, 34). CD1d belongs to the CD1 family with isoforms such as CD1a, CD1b, CD1c, and CD1e (35). CD1d is a transmembrane protein with two alpha-helices forming an antigen-presenting pocket above and a hydrophobic pocket below (28). This structure encapsulates hydrophobic portion of lipid antigens into the CD1d binding groove, and the polar portion of the antigen is exposed outside APCs to be recognized by T cell receptor (TCR) (28).

With an antigen-presenting molecule CD1d, adipocytes express high levels of lipid antigen loading and presentation-associated genes (28). There are two major pathways involved in antigen loading and presentation. The first one is endoplasmic reticulum (ER) and Golgi pathway, and the second one is endosomal and lysosomal pathway. In ER and Golgi pathway, the newly synthesized CD1d binds to *β*2-microglobulin in ER, and lipid antigens are loaded onto CD1d in Golgi by chaperone proteins, including microsomal triglyceride transfer protein (36, 37). Then, CD1d enters the transport step and fuses with the membrane to be exposed to cell surface of APCs. In endosomal and lysosomal pathway, CD1d is internalized in the form of endosome from plasma membrane. Chaperone protein and lipid transport protein replace low-affinity lipid antigens with high affinity lipid antigens (36, 37).

Although the clue for lipid antigen source has been suggested in several studies (38–41), the identity of endogenous lipid antigens in adipocytes has not been clearly elucidated. In the blood, circulating lipid metabolites are potentially subjected to behave as lipid antigens through scavenger receptor and very-low-density-lipoprotein receptor (VLDLR) (42). In VLDL-associated apoprotein APOE-deficient mice, the number of iNKT cells is altered (40). Also, fatty acid amide hydrolase enhances the presentation of lipid antigens by facilitating transport of serum lipids into APCs (41).

### Anti-Inflammatory Roles of Adipocytes *via* Lipid Antigen Presentation

**Figure 1:**
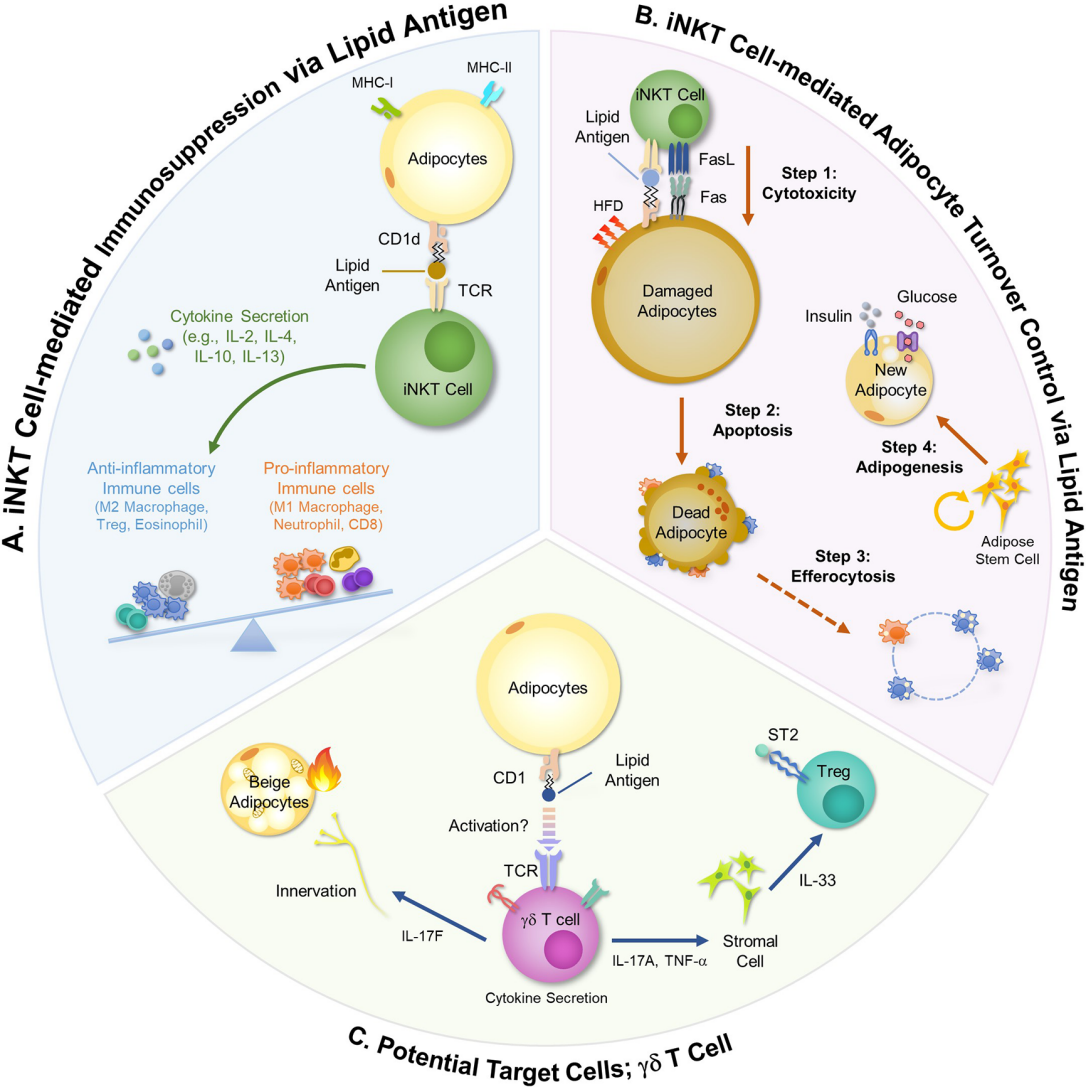
Immunomodulatory Roles of Adipocytes using Lipid Antigens. Adipocytes modulate activities of adipose immune cells *via* lipid antigen presentation. iNKT cells and *γδ* T cells are activated by lipid antigens and involve in the regulation of adipose tissue immunity and adipocyte functions. **(A)** In obesity, adipose iNKT cells activated by adipocyte-derived lipid antigens secret large amounts of anti-inflammatory cytokines such as IL-2, IL-4, IL-10, and IL-13. These cytokines stimulate Treg cells and polarize monocytes into anti-inflammatory M2 macrophages, thereby ameliorating pro-inflammatory responses in obese adipose tissue. **(B)** Adipose iNKT cells mediate hypertrophic and pro-inflammatory adipocyte death in obesity. Long-term HFD (over 8 weeks) upregulates CD95L (FasL) and CD95 (Fas) in adipose iNKT cells and damaged adipocytes, respectively. Interaction between CD95L and CD95 selectively stimulates damaged adipocyte death. After macrophage-mediated efferocytosis, adipose stem cells proliferate and *de novo* adipogenesis is promoted, leading to the generation of insulin-sensitive new adipocytes. **(C)** Given that *γδ* T cells recognize CD1-loaded lipid antigens, it has been suggested that adipocytes might regulate *γδ* T cell activity. *γδ* T cells secrete several cytokines such as IL-17 and TNF-α, controlling beige adipocyte formation and innervation. In addition, *γδ* T cells activate stromal cells to secrete IL-33, resulting in Treg cell recruitment.

The roles of CD1d in adipocytes have been investigated in genetically or diet-induced obesity models. Studies using adipocyte-specific CD1d knockout (CD1d^AKO^) mice have shown that adipocytes are crucial for the regulation of adipose iNKT cell activity (**Figure 1A**) (34, 43). In CD1d^AKO^ mice, the number of iNKT cells is decreased. Moreover, the levels of IL-4 secretion and FasL expression are downregulated in iNKT cells of CD1d^AKO^ mice compared to wild type (WT) mice, leading to aggravation in adipose tissue inflammation and insulin resistance (23, 34). The interaction between adipocytes and iNKT cells has been also examined in J*α*18 knockout (KO) mice and CD1d KO mice in which iNKT cells are deficient in whole body (24, 25). In the case of the above animal models lacking iNKT cells, body weight gain and adipocyte size are increased, and pro-inflammatory ATMs are more accumulated in obesity. Stimulation of iNKT cell activity by alpha-galactosylceramide (*α*-GC), a synthetic lipid antigen for iNKT cell and supplementation of iNKT cells into obese mice downregulate body weight gain and adipocyte size and upregulate secretion of anti-inflammatory adipokines. These metabolic changes are accompanied with restoration of insulin sensitivity (23, 25).

One of the major regulatory mechanisms for adipose tissue inflammation by adipose iNKT cell is through diverse cytokine secretion. For instance, adipose iNKT cells secrete IL-4 and IL-10 which promote M2 macrophage polarization (44). In obese mice, inhibition of IL-4/IL-10 signaling diminishes iNKT cell-dependent glucose homeostasis (25). Also, short-term HFD feeding induces the expression of arginase 1, one of the M2 marker genes, in adipose tissue of WT mice, but not in CD1d KO and IL-4 KO mice, indicating that adipose iNKT cells rapidly respond to HFD and produce IL-4 to suppress inflammatory responses *via* induction of M2 macrophages (45). Moreover, it has been shown that IL-2 secreted by adipose iNKT cells is involved in immunosuppressive function of Treg cells through promoting IL-10 production of Treg cells in adipose tissue (29). Upon short term HFD feeding, the number of adipose Treg cells is elevated in WT mice, but not in CD1d^AKO^ mice, underscoring the crucial roles of adipocyte CD1d in the regulation of the anti-inflammatory responses (33). Furthermore, it has been very recently reported that IFN*γ* produced by adipose iNKT cells in lean adipose tissue can serve to limit the expansion of ATMs by killing pro-inflammatory macrophages *via* NK cell stimulation (46).

These findings propose that activity control of iNKT cells by adipocytes and lipid antigens appears to be the key for adipose tissue immune balance (**Figure 1A**). In contrast, Satoh et al. has reported that adipose iNKT cells would exhibit pro-inflammatory characteristics by secreting IFN-*γ* because CD1d^AKO^ mice show adipose tissue inflammation and insulin resistance in obesity (43). Although there is no clear answer to explain opposite phenotypes in CD1d^AKO^ mice above, it has been suggested that these differences are probably due to different types of control mice (CD1d^flox/+^ *vs* CD1d^flox/flox^) and differences in high-fat diet (HFD) composition (tallow and safflower oil of high oleic type *vs* lard) (33). Moreover, it has been shown that adipose iNKT cells can be classified into several subpopulations that reveal either pro-inflammatory responses or anti-inflammatory responses (46), implying that characteristics of adipose iNKT cells might be affected by multilateral relationships between lipid antigen species and iNKT cell subtypes. Thus, it seems that veiled traits of adipose iNKT cells could be further uncovered when lipid antigens loaded on adipocytes and subtypes of adipose iNKT cells are identified in future studies.

### Adipocyte Turnover Control by Lipid Antigen(s)

Yearly, 10% of human adipocytes are dead and replaced with new adipocytes (47). Patients with cachexia, human immunodeficiency virus (HIV) or lipodystrophy syndrome show drastic loss of adipocytes (48–51). In obese mice, dead adipocytes are frequently found in epididymal adipose tissue (23, 52). Although adipocyte death is associated with adipose tissue inflammation in obesity, the causal factors that would induce adipocyte death have not been fully elucidated. Recently, it has been reported that, in hypertrophic adipocytes, the expression of Fas (CD95) is upregulated and is positively correlated with the degree of adipocyte death (**Figure 1B**) (23). Apoptotic pathway is induced in Fas-positive cells when Fas is bound to FasL (53). In obese adipose tissue, the portion of FasL-positive iNKT cells is significantly elevated, but not in CD4 and CD8 T cells, indicating that iNKT cells would be a major killer cell type to induce hypertrophic adipocyte death in obesity (23). Through *in vitro* and *in vivo* experiments, it has been shown that hypertrophic adipocytes with pro-inflammatory characteristics stimulate iNKT cells by lipid antigen presentation *via* CD1d (23). Then, the activated iNKT cells selectively kill hypertrophic and pro-inflammatory adipocytes (23). iNKT cell-mediated hypertrophic adipocyte death is consistently observed in both diet-induced obese mice and genetically obese *db/db* mice (23). After iNKT cell-mediated adipocyte death, adipocyte stem cells proliferate and differentiate into new and small adipocytes exhibiting elevated insulin sensitivity (**Figure 1B**) (23, 54). Together, it has been suggested that, in obesity, activity control of iNKT cells by adipocytes is crucial for adipocyte turnover, contributing to the improvement of insulin sensitivity.

### Adipocyte Death and Adipose Tissue Inflammation

Although adipocyte death and ATMs surrounding dead adipocytes are frequently observed in obesity, the relationship between adipocyte death and inflammation remains elusive. Activation of iNKT cells by *α*-GC administration into HFD-fed obese mice induces apoptosis of hypertrophic adipocytes, accompanied by the increase in the portion of M2 macrophages compared to that of M1 macrophages (23). Similarly, the number of CD206 and CD301-positive M2-macrophages increases when adipocyte-specific apoptosis is induced in FAT-ATTACK mice (55). It seems that transient induction of apoptosis in adipocytes would upregulate anti-inflammatory responses. On the other hand, continuous adipocyte death resulted from chronic inflammation or deficiency of key enzymes involved in sphingolipid synthesis and mevalonate pathway often causes systemic pro-inflammatory responses (56, 57). Furthermore, if apoptotic cells are not rapidly and properly cleared by efferocytosis, the membrane of apoptotic cells is ruptured and transformed into necrosis-like cells, provoking inflammation. Thus, it is likely that controversial results of adipocyte death on adipose tissue inflammation would be due to several factors: whether types of adipocyte death are apoptotic or necrotic, whether adipocyte death is transient or persistent, and whether debris of dead adipocytes are well cleared.

The clearance of apoptotic cells by professional and non-professional phagocytes is essential for maintenance of tissue homeostasis (58). In response to apoptotic cells, macrophages suppress production of pro-inflammatory cytokines and enhance secretion of molecules that dampen inflammation, and mediate resolution and repair. Thus, defective efferocytosis leads to inflammation and impaired resolution, underlying various chronic inflammatory diseases such as atherosclerosis, obesity, diabetes, cardiovascular diseases, and cancer (58). In obese mice, macrophages appear to exhibit impaired efferocytosis, which is associated with higher number of apoptotic cells and greater expression of pro-inflammatory cytokines within wounds (59, 60). It has been proposed that defects of omega-3 fatty acids, erythropoietin, and MER proto-oncogene tyrosine kinase would suppress efferocytosis of dying/dead cells in atherosclerotic lesions, skin, and heart in obesity (58). However, to date, most studies have not focused on clearance of dead adipocytes, although dead adipocytes and ATMs surrounding them are abundantly observed in obesity. Future studies are required to unravel complex relationships between adipocyte death, efferocytosis, and adipose tissue inflammation.

### *γδ* T Cells: Potential Target Cells of Adipocytes

*γδ* T cell is one of the innate lymphocytes that are not restricted to MHC molecules but recognize CD1 molecules. In adipose tissue, *γδ* T cells exhibit resident characteristics and occupy 5–15% of total T cells (26). Upon HFD, the number of *γδ* T cells increases and they promote accumulation of pro-inflammatory macrophages, worsening adipose tissue inflammation and insulin resistance (30). In contrast, it has been shown that IL-17A-producing *γδ* T cells are involved in the maintenance of adipose Treg population by promoting secretion of IL-33 from stromal cells, contributing to suppression of adipose tissue inflammation (**Figure 1C**) (26). In addition, under short term ketogenic diet (KD) which contains high fat and low carbohydrate, *γδ* T cells suppress adipose tissue inflammation and protect metabolic dysregulation through increasing expression of genes related to tissue repair (61). Conversely, long-term KD drastically decrease the number of *γδ* T cells and aggravates obesity and glucose intolerance (61). Although it remains to be clarified whether adipose *γδ* T cells would upregulate or downregulate inflammatory responses in adipose tissue, it seems that *γδ* T cell could play certain roles in inflammatory responses in adipose tissue. In addition to the regulation of adipose tissue inflammation, *γδ* T cells modulate adipocyte functions such as lipolysis and thermogenesis (26). In brown and subcutaneous adipose tissue, *γδ* T cells boost thermogenic programs by stimulating IL-33 secretion in stromal cells or promoting innervation in adipose tissue (**Figure 1C**) (26, 27). Given that *γδ* T cells could recognize lipid antigens loaded on CD1 family, it is plausible to speculate that adipocytes would function as potential APCs in adipose tissue.

## Relationship Between Lipid Metabolism in Adipocytes and Adipose Tissue Immunity

In adipose tissue, lipid metabolism is dynamically regulated upon diverse physiological conditions such as fasting, HFD, and aging. If lipid metabolism is dysregulated in adipocytes due to environmental or genetic factors, adipose tissue immunity and whole body energy metabolism are distorted. It has been suggested that endogenous lipids such as free fatty acids (FFAs) and eicosanoids modulate innate and adaptive immune cells (62). Furthermore, HFD provokes uncontrolled basal lipolysis and promotes unnecessary release of FFAs, causing imbalanced immune responses in adipose tissue. Also, when lipid storage capacity of adipocytes is defective by ablation of lipid droplet (LD) binding proteins such as Perilipin1 (Plin1), the levels of triglyceride and FFAs are elevated in adipose tissue and serum, which is accompanied by adipose tissue inflammation and insulin resistance (63). In this section, we cover how adipocytes regulate adipose immune responses by controlling lipid metabolism.

### Regulation of Adipose Immune Responses by Lipid Metabolites

Lipid metabolites are associated with numerous human diseases, including atherosclerosis, rheumatoid arthritis, and other inflammation-linked metabolic diseases (64). While it has been considered for a long time that lipid metabolites are key energy sources, the importance of lipid metabolites as signaling molecules has been accumulated (65–67). Eicosanoids, certain FFAs, and FFA derivatives are able to act as signaling molecules in the regulation of immune responses (64). Among them, several lipid metabolites are produced by adipocytes or adipose tissues (19–21). Palmitoleate (C16:1n7), a long-chain monounsaturated FA, is produced through *de novo* lipogenesis in adipose tissue and downregulates pro-inflammatory gene expressions in macrophages (68–71). Also, in adipocytes, palmitic acid esters of hydroxy stearic acids (PAHSAs) synthesized by carbohydrate response element binding protein (ChREBP) regulate adipose tissue inflammation. While adipocyte-specific ChREBP knockout (ChREBP^AKO^) mice exhibit decreased PAHSA levels and increased ATMs in adipose tissue, PAHSA administration ameliorates pro-inflammatory responses in adipose tissue of ChREBP^AKO^ mice (72).

**Figure 2:**
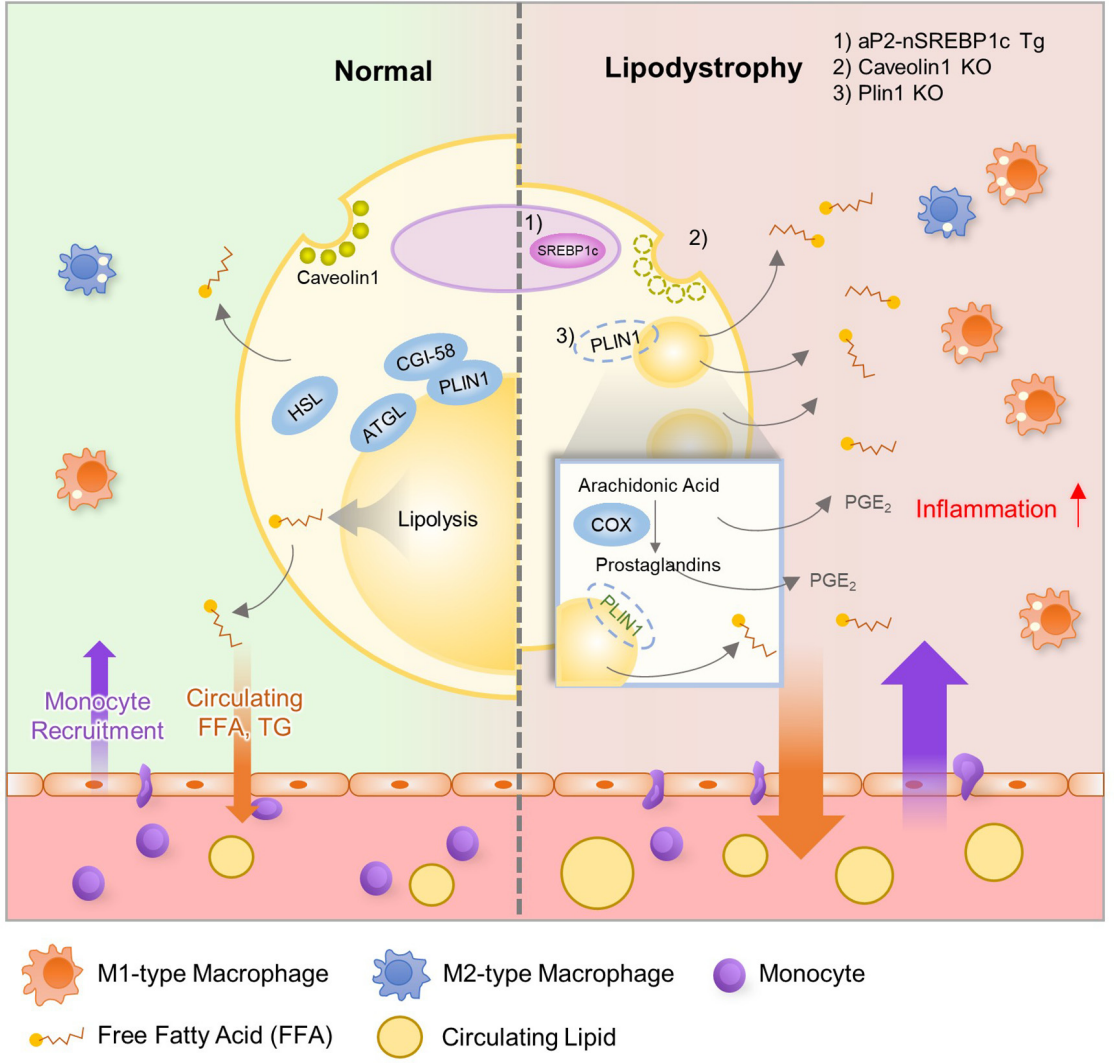
Relationship between Lipodystrophy and Adipose Tissue Inflammation. In adipocytes, lipid metabolism is well balanced by several genes, including Srebp1c, Atgl, Hsl, Cgi-58, Plin1, and Fsp27. However, lean subjects with lipodystrophy show dysregulated lipid metabolism with increased inflammation and insulin resistance. Evidence suggests that dysregulation of lipid metabolism could influence adipose tissue inflammation in lipodystrophy. aP2-nuclear form of SREBP1c transgenic (aP2-nSREBP1c Tg) mice and Caveolin1 KO mice show significantly reduced fat mass and display metabolic dysregulation including insulin resistance and dyslipidemia. In addition, Plin1 deficiency induces partial fat loss, leakage of FFAs, ATM accumulation, dyslipidemia and systemic insulin resistance. In these lipodystrophic models, several lipid metabolites such as FFA and PGE2 recruit monocytes into adipose tissue and worsen adipose tissue inflammation.

In addition to *de novo* lipogenesis, certain lipid metabolites which regulate adipose tissue inflammation are produced by lipolysis. Recently, it has been shown that Plin1 inhibits futile prostaglandin secretion to restrict pro-inflammatory responses in adipose tissue (63). Plin1 deficiency in adipocytes impairs lipid storage into LDs and stimulates lipolysis, causing adipose tissue loss and unnecessary leakage of pro-inflammatory lipid metabolites. In adipose tissue of Plin1 KO mice (**Figure 2**), pro-inflammatory gene expression and M1-type ATM accumulation are increased. Suppression of lipolysis by knockdown or inhibition of lipases attenuates the effects of Plin1-deficient adipocytes on monocyte migration. Moreover, lipidomic analysis and administration of cyclooxygenase inhibitor indicate that enhanced adipose tissue inflammation is mediated by excessive prostaglandin E_2_ (PGE_2_) secretion in Plin1-deficient adipocytes (62). Thus, it has been proposed that reducing futile lipolysis in adipocytes could downregulate adipose tissue inflammation through the control of pro-inflammatory lipid metabolite secretion (63).

Circulating FFAs are elevated in obesity and lipodystrophy, which is closely related to metabolic disorders including type 2 diabetes and atherosclerosis. FFAs including palmitic acids are able to activate inflammatory responses and also used to produce ceramides. Ceramides are one of important metabolites whose levels are elevated in obesity (73). Increased ceramides contributes to adipose tissue inflammation and dysregulation of energy homeostasis. In macrophages, ceramide initiates p38 MAPK and JNK signaling pathways, polarizing ATMs towards M1 macrophages (74). Moreover, ceramides activate NLR family pyrin domain containing 3 (NLRP3) inflammasome and promote secretion of IL-1β and IL-18 in macrophages, aggravating adipose tissue inflammation and glucose intolerance in obesity (75).

### Lipodystrophy and Adipose Tissue Inflammation

Although lipodystrophy and adipose tissue expansion such as obesity are somewhat opposite in terms of adipose tissue mass, both pathological states often exhibit similar metabolic dysregulation (76–78). Obesity-induced low-grade and chronic inflammation is one of the major factors to promote insulin resistance (12, 79). Also, severely lean patients with lipodystrophy or cachexia reveal enhanced inflammation with insulin resistance even though underlying mechanisms are not fully uncovered. Nonetheless, it has been suggested that immune responses in adipose tissue could be involved in the development of insulin resistance in lipodystrophy (80, 81). Pro-inflammatory gene expression and ATM accumulation are promoted in adipose tissue of lipodystrophic animal models even with less adipose tissue mass. For instance, aP2-nuclear form of sterol regulatory element-binding protein 1c (SREBP1c) transgenic (aP2-nSREBP1c Tg) mice and Caveolin1 KO mice show significantly reduced fat mass and display metabolic dysregulation including insulin resistance and dyslipidemia (82–84). In these lipodystrophic models, increases in pro-inflammatory cytokine and ATM accumulation are observed in adipose tissue (**Figure 2**) (84). In addition, Plin1 deficiency reveals partial fat loss, ATM accumulation, dyslipidemia and systemic insulin resistance in both mouse and human (63, 85). In aP2-nSREBP1c Tg mice, anti-inflammatory strategies such as salicylate treatment or crossing with myeloid cell-specific I*κ*B kinase (IKK*β*) KO mice do not ameliorate insulin resistance (83). On the other hand, in Plin1 KO mice, macrophage depletion by clodronate treatment or inhibition of synthesis of pro-inflammatory lipid metabolites in adipocytes mitigates systemic insulin resistance (63). These results indicate that the precise relationship between adipose tissue inflammation and systemic energy homeostasis remains to be thoroughly elucidated under lipodystrophic conditions.

### Aging-Related Decrease in Lipolysis

Aging is a chronic and complex physiological process that gradually deteriorates energy homeostasis (86). Dysfunction of adipose tissue is one of the major factors to provoke aging-related metabolic disorders including type 2 diabetes and cardiovascular diseases. In the elderly, the processes of lipolysis and lipid storage in adipose tissue are not properly controlled. As a result, mobilization of FFAs is dysregulated, causing visceral adiposity, lower exercise capacity, and cold intolerance. These alterations of adipose tissue are closely associated with adipose tissue immunity (87). Adipose macrophages and B cells are involved in age-related reduction of lipolytic activity. In aged mouse model, macrophages degrade catecholamine in a NLRP3 inflammasome-dependent manner in adipose tissue, driving lipolysis resistance in adipocytes (88). When NLRP3 inflammasome is activated in aged macrophages, the expression of monoamine oxidase (MAOA) which is known to degrade noradrenaline is increased by growth differentiation factor-3 (88). Moreover, aging stimulates expansion of adipose B cells in fat-associated lymphoid clusters (FALC), which is mediated by activation of NLRP3 inflammasome and IL-1 signaling (89). It has been shown that inhibition of MAOA in macrophages or depletion of B cell reverses the age-related decline in lipolysis and restore age-associated adipose tissue impairment (89). However, in human adipose tissue, the major cell type expressing MAOA is different from mice. In human adipose tissue, MAOA is mainly expressed in mature adipocytes, unlike mice, contributing to aging-associated reduction in lipolysis (90).

## The Novel Roles of Adipose Stem Cells in The Regulation of Adipose Tissue Immunity

ASCs are composed of heterogeneous populations and each population has unique characteristics. ASCs are largely divided into adipogenic and non-adipogenic subtypes (91). Adipogenic ASCs preferentially differentiate into adipocytes in response to excess energy, which increases energy storage capacity of adipose tissue. This process, called hyperplasia, mediates healthy adipose tissue expansion and attenuates adipose tissue inflammation in obesity. On the other hand, non-adipogenic ASCs secrete various pro- and anti-inflammatory cytokines, lipokines, and collagens, which could affect activity and recruitment of adipose immune cells. In addition, it appears that non-adipogenic ASCs would be key players for distinct immune responses between subcutaneous white adipose tissue (sWAT) and visceral white adipose tissue (vWAT). As the roles of adipogenic ASCs have been well discussed in previous reviews (92, 93), we cover the novel roles of non-adipogenic ASCs in the regulation of adipose tissue immunity.

### Novel Roles of ASCs in the Regulation of Adipose Tissue Immunity

Adipose tissue is divided into adipocyte and stromal vascular cell (SVC) fraction, and SVC fraction is further classified into ASCs (CD45^-^CD31^-^), immune cell (CD45^+^), endothelial cell (CD31^+^), and red blood cell. In the last several years, single cell RNA-sequencing (scRNA-seq) has been used to reveal subpopulation and characteristics of ASCs, providing compelling evidence that ASCs would exhibit molecular heterogeneity and functional diversity (94, 95). Interestingly, it has been proposed that ASCs not only have adipogenic potential, but also exhibit anti-adipogenic and immunomodulatory roles (96).

**Figure 3:**
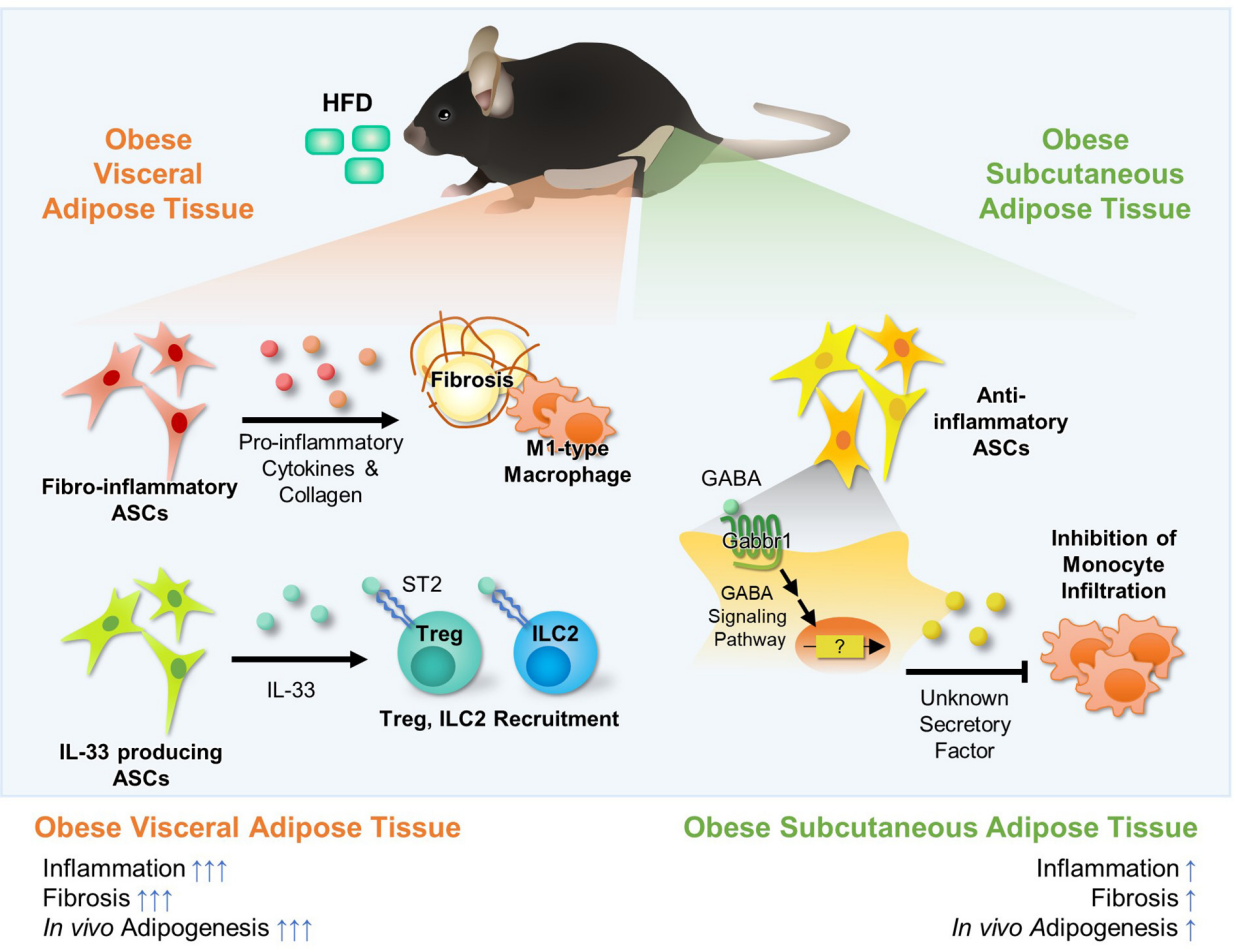
Fat Depot-specific Roles of Adipocyte Stem Cells (ASCs) in the Regulation of Adipose Tissue Immunity. White adipose tissues consist of major two fat depots; visceral adipose tissue and subcutaneous adipose tissue. These two fat depots exhibit several differences in inflammatory responses, fibrosis, and adipogenesis. ASCs are major cell types comprising of adipose tissue, and they are largely divided into adipogenic and non-adipogenic clusters. In visceral adipose tissue, there are fibro-inflammatory ASCs (lin−Pdgfr*β*+Ly6c+ cells or lin−Pdgfr*α*+Gp38+CD9+). The number of fibro-inflammatory ASCs increases in obesity and they secret pro-inflammatory cytokines (*e.g.*, IL-6, Ccl2) and ECM components (*e.g.*, Col1a1, Col3a1), promoting fibrosis. Moreover, it has been reported that IL-33 producing non-adipogenic ASCs (lin−Pdgfr*α*+PPAR*γ*−) are involved in recruitment of Treg and ILC2 *via* IL-33 secretion, which suppresses inflammation in visceral adipose tissue. Recently, it was reported that, in subcutaneous adipose tissue, ASCs (CD31−CD34+Sca1+) suppress monocyte infiltration, which is potentially regulated by GABA signaling. However, the secretory factors that inhibit monocyte infiltration in subcutaneous adipose tissue have not been elucidated yet.

ASCs secrete pro-inflammatory cytokines (*e.g.*, IL-6, IL-8, IL-11, TNF-α), anti-inflammatory cytokines (*e.g.*, TGF-*β*, IL-10), growth factors, chemokines (Cxcl5), and lipokines (PGE2) (97). Upon HFD, the number of fibro-inflammatory stem cells (lin^−^Pdgfrβ^+^Ly6c^+^ cells, lin^−^Pdgfrα^+^Gp38^+^CD9^+^) is upregulated and they highly express pro-inflammatory cytokines (*e.g.*, IL-6, Ccl2, Cxcl2, Cxcl10) and extracellular matrix components (*e.g.*, Col1a1, Col3a1), causing adipose tissue inflammation (**Figure 3**) (98–100). In human and mouse, CXCL1^+^ mesothelial cells (CD45^-^CD31^−^Ter119^−^CD41^−^PDPN^+/−^) recruit neutrophils into the FALC *via* protein arginine deiminase 4 during peritonitis and promote the aggregation of neutrophils, providing first layer of immunological defense in vWAT (101). On the other hand, another population of ASCs that suppress adipose tissue inflammation has been also reported (102–104). Lin^-^Pdgfrα^+^Sca1^+^ population is a major source of IL-33 in vWAT (**Figure 3**) (102). IL-33^+^ ASCs recruit anti-inflammatory Treg and ILC2 cells in lean subjects, contributing to suppression of adipose tissue inflammation (102).

It has been shown that ASCs would be the key cell type that explains distinct inflammatory patterns between sWAT and vWAT in obesity (**Figure 3**) (100, 103, 104). In obese mice, vWAT shows the higher number of infiltrated macrophages and crown-like structures, whereas sWAT is less prone to inflammation. However, it is still unknown which factors make the differences in inflammatory responses between the two major fat depots in obesity. Very recently, it has been demonstrated that SVCs of sWAT secrete certain factors to repress monocyte recruitment, and that transplantation of ASCs derived from sWAT into vWAT suppresses ATM infiltration in vWAT (103, 104). Interestingly, gamma-aminobutyric acid (GABA) signaling is one of the most differentially expressed pathways between sWAT and vWAT in obesity. In HFD-induced obese mice, GABA treatment inhibits ATM infiltration in sWAT-selective manner, but not in vWAT (102). Thus, it has been proposed that GABA signaling in ASCs might be one of the potential pathways that could selectively suppresses inflammatory responses in sWAT (103).

Given that ASCs have high proliferation rate, adipogenic potential, and immunomodulatory roles, they have been considered therapeutic target for recovery of adipose tissue homeostasis. Recently developed scRNA-seq analysis dissects ASCs into three or more subpopulations with their own distinct functions. Proliferative and stem cell-like ASCs can be used in tissue repair and regenerative processes. Adipogenic and anti-adipogenic subpopulations of ASC can increase or decrease buffering capacity of adipose tissue, respectively. In addition, ASCs that exhibit immunomodulatory properties can be used to control inflammatory responses of adipose tissues. Although complicated networks between ASCs and adipose tissue constituent cells need to be further investigated, recent approaches equipped with high techs would provide new therapeutic targets against adipose tissue dysfunction, particularly, in obesity.

## Limitations and Future Directions

There are several points to be solved in future studies. First, it remains elusive which kinds of endogenous lipid antigens would be presented by adipocyte CD1d in obesity. Even though *α*-GC has been used as an activator for iNKT cells, *α*-GC is an exogenous and quite potent activator, which might be different from patho-physiologic conditions. Second, it is required to identify antigen presenting cells and lipid antigens that regulate the activity of γδ T cells in adipose tissue. Third, the mechanisms of ATM recruitment by lipid metabolites such as PGE_2_ should be elucidated in future studies. Lastly, while recent technical advances (e.g., scRNA-seq) have proposed novel subpopulations of adipocytes and discovered new relationships between adipocyte subpopulations and immune cells, it remains to be validated with proper *in vivo* models (105–108). Also, there are still huge technical obstacles in the analysis of lipid profiles from each adipocyte subpopulations as well as immune cells.

## Conclusion

Lipids are key energy sources and primary building blocks for plasma membranes and intracellular organelles. Moreover, lipid metabolites participate in numerous signal transduction and regulate multiple cellular functions. Recently, it has been suggested that lipid metabolites are crucial bioactive molecules in immune system (18–20). Here, we have discussed the immunomodulatory roles of lipid metabolites of adipocytes upon metabolic stimuli. In response to altered metabolic environments, adipocytes sensitively and dynamically control lipid metabolism and present or secrete lipid metabolites to modulate characteristics of adipose immune cells. Thus, it is plausible to speculate that adipocytes not only use lipid metabolites to maintain their structures and functions, but also actively utilize lipid metabolites as key messengers to communicate with adipose immune cells. The interplay between adipocytes and adipose immune cells leads to fine-tuning adipose tissue immunity and adipose tissue remodeling, which eventually contributes to maintenance of systemic energy metabolism. Nonetheless, there are remaining issues to be solved in future studies. For instance, the lipid antigen presented by adipocytes and lipid metabolites secreted by adipocytes are not fully identified. There have been technical difficulties such as extraction of lipids, identification of specific lipid species, and quantitation of the vast array of lipids. Thus, solving these issues will enhance our insights about the mechanisms by which adipocytes govern adipose tissue immunity, and further suggest new therapeutic approaches on metabolic complications caused by adipose tissue inflammation.

## Author Contributions

JP, JHS, SMH, YJP, JYH, SSC, and JBK contributed to the writing of the manuscript under JK’s supervision. All authors contributed to the article and approved the submitted version.

## Funding

This work was supported by the National Research Foundation of Korea (NRF) grant funded by the Korea government (MSIT) (No. NRF-2020R1A3B2078617).

## Conflict of Interest

The authors declare that the research was conducted in the absence of any commercial or financial relationships that could be construed as a potential conflict of interest.

## Abbreviation

α-GC, Alpha-galactosylceramide; APC, Antigen presenting cell; ASC, Adipose stem cell; ATM, Adipose tissue macrophage; CD1dAKO, Adipocyte-specific CD1d depletion; ChREBP, Carbohydrate response element binding protein; ER, Endoplasmic reticulum; FALC, Fat-associated lymphoid cluster; FFA, Free fatty acid; GABA, Gamma-aminobutyric acid; HFD, High-fat diet; HIV, Human immunodeficiency virus; IFN, Interferon; IKKβ, IkB kinase; IL, Interleukin; ILC, Innate lymphoid cell; iNKT, Invariant natural killer T; KD, Ketogenic diet; KO, Knock out; LD, Lipid droplet; MAOA, Monoamine oxidase; NLRP3, NLR family pyrin domain containing 3; NO, Nitric oxide; PAHSA, Palmitic acid esters of hydroxy stearic acid; PGE2, Prostaglandin E2; Plin, Perilipin; scRNA-seq, Single cell RNA-sequencing; SREBP1c, Sterol regulatory element-binding protein 1c; SVC, Stromal vascular cell; sWAT, Subcutaneous white adipose tissue; TCR, T cell receptor; TNF, Tumor necrosis factor; Treg, Regulatory T cell; VLDLR, Very-low-density-lipoprotein receptor; vWAT, Visceral white adipose tissue; WAT, White adipose tissue.
